# How Perceptions of HIV-Related Stigma Affect Decision-Making Regarding Childbirth in Rural Kenya

**DOI:** 10.1371/journal.pone.0051492

**Published:** 2012-12-12

**Authors:** José S. Medema-Wijnveen, Maricianah Onono, Elizabeth A. Bukusi, Suellen Miller, Craig R. Cohen, Janet M. Turan

**Affiliations:** 1 University Medical Centre Groningen, University of Groningen, Groningen, The Netherlands; 2 Department of Obstetrics, Gynecology, and Reproductive Sciences, University of California San Francisco, San Francisco, California, United States of America; 3 Research Care and Training Program, Center for Microbiology Research, Kenya Medical Research Institute (KEMRI), Nairobi, Kenya; 4 Center of Expertise in Women’s Health & Empowerment, University of California Global Health Institute, San Francisco, California, United States of America; 5 Department of Health Care Organization and Policy, School of Public Health, University of Alabama, Birmingham, Birmingham, Alabama, United States of America; University of Ottawa, Canada

## Abstract

**Introduction:**

HIV prevalence among pregnant women in Kenya is high. Furthermore, there is a high risk of maternal mortality, as many women do not give birth with a skilled healthcare provider. Previous research suggests that fears of HIV testing and unwanted disclosure of HIV status may be important barriers to utilizing maternity services. We explored relationships between women’s perceptions of HIV-related stigma and their attitudes and intentions regarding facility-based childbirth.

**Methods:**

1,777 pregnant women were interviewed at their first antenatal care visit. We included socio-demographic characteristics, stigma scales, HIV knowledge measures, and an 11-item scale measuring health facility birth attitudes (HFBA). HFBA includes items on cost, transport, comfort, interpersonal relations, and services during delivery at a health facility versus at home. A higher mean HFBA score indicates a more positive attitude towards facility-based childbirth. The mean HFBA score was dichotomized at the median and analyses were conducted with this dichotomized HFBA score using mixed effects logit models.

**Results:**

Women who anticipated HIV-related stigma from their male partner had lower adjusted odds of having positive attitudes about giving birth at the health facility (adjusted OR = .63, 95% CI 0.50–0.78) and less positive attitudes about health facility birth were strongly related to women’s intention to give birth outside a health facility (adjusted OR = 5.56, 95% CI 2.69–11.51).

**Conclusions:**

In this sample of pregnant women in rural Kenya, those who anticipated HIV-related stigma were less likely to have positive attitudes towards facility-based childbirth. Furthermore, negative attitudes about facility-based childbirth were associated with the intention to deliver outside a health facility. Thus, HIV-related stigma reduction efforts might result in more positive attitudes towards facility-based childbirth, and thereby lead to an increased level of skilled birth attendance, and reductions in maternal and infant mortality.

## Introduction

There is evidence that HIV and maternal mortality are not separate, but rather two intersecting epidemics in sub-Saharan Africa, as HIV is an increasing contributor to direct and indirect causes of maternal death [1]. Childbirth with a skilled healthcare provider is one of the key strategies recommended for prevention of maternal mortality and morbidity and has also been identified as important for enhancing prevention of mother-to-child transmission (PMTCT) of HIV [2]. In Kenya, only 43% of women give birth in a health facility with a skilled healthcare provider [3] and the lifetime risk of maternal mortality is 1 in 38 [4]. Many Kenyan women deliver outside the health facility with the assistance of an untrained traditional birth attendant (TBA).

A significant body of research has focused on the identification of factors that might explain why many women deliver outside the health facility globally. Investigators have found the place of delivery to be associated with socio-demographic factors; including age, education, religion, parity, and income[3], [5]–[15]. Barriers for delivering at a health facility include lack of transport, long travel times, and lack of money or mandatory supplies[6], [7], [16]–[18]. Furthermore, home birth is highly valued in many African cultures and delivering outside the homestead may be a cultural taboo [11], [15].

Another potentially important factor in decision-making about the type of assistance during childbirth may be the perceived quality of maternity care [8]. In fact, researchers in Ethiopia found that perceived quality of care was more important than distance and cost in decision-making for the place of delivery [19].

One factor potentially affecting women’s perceptions of maternity services that has not been examined in previous research is HIV-related stigma. Fears of stigma, discrimination, and lack of confidentiality in health facilities have been shown to discourage people from accepting HIV testing and linking to HIV care after receiving an HIV-positive test result in multiple settings around the world [20]. Our previous qualitative research in Kenya also identified fear of HIV testing as a reason that some women may avoid childbirth in health facilities, as they fear unwanted disclosure of their HIV testing and HIV status to their spouse or family at the health facility, and subsequent stigma and discrimination [21]. In-depth interviews with healthcare workers also revealed that some pregnant women experience stigma and discrimination from maternity workers [22]. Other literature from Kenya also reveals that women may perceive that they will be forced to test for HIV when visiting maternity services [23], as well as having concerns about the confidentiality of their HIV status at health facilities [24].

We investigated the relationships between pregnant women’s perceptions of HIV-related stigma, their attitudes towards giving birth at the health facility, and their intended type of assistance during birth. We hypothesized that anticipation of HIV-related stigma would lead to less positive attitudes towards delivery at the health facility and thereby would lead to an intention to deliver outside the health facility.

## Methods

### Ethical Statement

This study received ethical approval from the Kenya Medical Research Institute (KEMRI) Ethical Review Committee and the Committee on Human Research of the University of California, San Francisco.

### Setting and Participants

The Maternity in Migori and AIDS Stigma (MAMAS) Study was conducted at sites supported by Family AIDS Care and Education Services (FACES), an HIV prevention, care, and treatment program operating in Nyanza Province Kenya. HIV prevalence among women of reproductive age in Nyanza Province is estimated at 16% [3]. Women at least 18 years old in their first seven months of pregnancy, who were visiting the antenatal care clinic (ANC) for the first time in their pregnancy and did not know their current HIV status (never tested or tested negative more than 3 months ago) were recruited at nine governmental health facilities. After obtaining signed informed consent, participants were interviewed in their preferred language (Dholuo, Kiswahili, or English) by a trained interviewer. Subsequently all women were offered voluntary HIV counselling and testing during the ANC visit, followed by post-test counseling and antiretroviral drugs for PMTCT for those who tested HIV-positive, as per Kenyan national guidelines [25]. Information on their acceptance of HIV testing and their test result were subsequently obtained from the women’s medical charts. Recruitment, baseline interviews and HIV testing took place between November 2007 and April 2009.

### Measures

#### Stigma measures

To measure perceptions of HIV-related stigma at baseline, two stigma scales developed in sub-Saharan Africa were included in the survey. **Anticipated stigma** is the anticipation that one will personally experience specific types of stigma or discrimination if one is found to be HIV-positive and others learn of one’s HIV status. This type of stigma was measured by a nine-item scale originally developed in Botswana that also captures from whom they expected this stigma: male partner (break-up of relationship, physical abuse), family members (neglect, denial of care), or others (treatment as outcast, bad treatment at school or work, bad treatment by health workers, loss of friends, loss of job) [26]. Researchers in Botswana found this scale to have high internal reliability consistency (Cronbach’s alpha = 0.77) [26]. In the current study, a total anticipated stigma score was calculated by taking the mean of all responses for women who provided responses to at least 6 of the 9 scale items, and women who provided 5 or fewer responses were coded as missing. We furthermore created dichotomous measures of anticipated stigma for the different ‘sources’ of stigma. Given our previous analyses showing the importance of male partner stigma, as compared to other dimensions of stigma, as a predictor of HIV test refusal in this population [27], we chose this as our primary anticipated stigma variable. Analyses were conducted using all of the different anticipated stigma measures, but we chose to construct a more parsimonious model including the anticipated male stigma variable, which had the strongest relationship with the outcome.

To measure general attitudes and perceptions about persons living with HIV (PLWH) and how they are treated in their community, referred to as **perceived community stigma** in this paper [27], [28], we used a 22-item scale developed by Genberg et al. [29], [30] with items like ‘People living with HIV/AIDS deserve to be punished’ and ‘People living with HIV/AIDS in this community face rejection from their peers’. This scale was found to have high internal consistency validity and good divergent validity in both Thailand and Zimbabwe [30]. As reported elsewhere [27], internal-consistency reliability of these scales in our sample of pregnant women in Kenya was high (Cronbach’s alpha.86 for the anticipated stigma scale and.85 for the perceived community stigma scale).

#### Health facility birth attitude (HFBA) measure

To measure women’s perceived quality of care and attitudes towards giving birth at the health facility, we developed an 11-item scale derived from our previous qualitative research in Nyanza Province. In that study, we identified common beliefs and attitudes towards giving birth at the health facility assisted by a skilled healthcare provider versus giving birth at home assisted by a TBA, based on in-depth interviews with postpartum women, male partners, TBAs, and health workers [31]. This resulted in 11 statements with which women were asked to agree or disagree (coded 1 or 0). A total HFBA score was calculated by taking the mean score for women who provided responses to 8 or more scale items, and women who provided 7 or fewer responses were coded as missing, with a higher mean score indicating a more positive attitude towards giving birth at a health facility. The internal-consistency reliability of this score in this sample was found to be moderate (Cronbach’s α = .60), but acceptable for research in the early stages [32].

#### Other variables

The survey also included questions on socio-demographic characteristics. Questions that assessed knowledge regarding HIV transmission and prevention (for example, can a person get HIV by sharing a meal with someone who is infected?) were included and were used to construct a HIV knowledge index based on the number of items that were answered correctly. The primary outcome variables for our analyses of women’s intentions regarding childbirth were based on questions in which participants were asked where they intended to give birth and with what type of assistance. Participants were also asked about expected costs related to their delivery (transportation, supplies, medicines, fees). We considered giving birth in a health facility to be equivalent to giving birth with a skilled health care provider (although recognizing that the quality of birth attendance provided in health facilities may not be optimal) [33].

### Statistical Analysis

Initial analyses were conducted using SPSS 16.0. [34]. With HFBA as the dependent variable, initial tests of bivariable associations were conducted using the Pearson’s Chi-square for nominal or ordinal variables, student T-test and ANOVA for continuous variables, and Kruskal-Wallis-test or the Mann-Whitney U-test for continuous variables with non-normal distributions. Following these initial analyses, HFBA was dichotomized into high and low, with the median (.73) chosen as a cut-off point. After conducting unadjusted logistic regression analyses to identify significant associations, we ran a mixed-effects logit model [35] using Stata 11 [36]. This multivariable model accounted for clustering by site and included variables that were significantly associated with the HFBA score in bivariable analyses (p<.05), as well as other variables that have been shown to be important for childbirth decision-making in the literature. Specifically, we included socio-economic factors including age, educational level, and occupation, as these factors related to women’s empowerment have been shown to be important factors in decision-making regarding childbirth in Kenya and similar countries [3], [37]. These analyses were repeated with a continuous version of the HFBA variable (normally transformed) to examine the potential impact of dichotomization on our results. Similar analytic methods were used to examine the predictors of intended type of assistance during delivery, in that variables found to have statistically significant relationships with the outcome in bivariable analyses (p<.05) were included in the multivariable model (education, occupation, being in a polygamous relationship, HFBA, expectations to pay for transport or supplies, and anticipated stigma), as well as household television ownership as a measure of wealth and access to mass media [38]. Binomial regression was used to obtain estimates of risk ratios for the multivariable analyses, with adjustment for clustering by site.

Finally, we conducted exploratory mediation analyses to examine if the effects of anticipated male partner HIV-related stigma on intended type of delivery might potentially operate through effects on HFBA. The conditions for mediation were met [39] as: 1) anticipated male partner stigma is significantly associated with HFBA, 2) HFBA independently predicts intended type of delivery assistance, and 3) the association of anticipated male partner stigma with intended type of birth assistance is reduced after adjustment for HFBA.

## Results

### Study Sample

Baseline interview data were available for 1,777 women (Table 1). Per Kenyan National Guidelines all women should have been offered HIV testing; however, no testing services were available for 203 (12%) of participants. During the study period, HIV testing services were sometimes unavailable at the study sites due to lack of HIV test kits, health worker absence, or other health system failures. Of the women for whom testing was available, 1,431 (94%) agreed to be tested, and 259 (18% of those tested) tested HIV-positive. Indicators of the women’s anticipations of stigma if they were to test positive for HIV are also presented in Table 1.

**Table 1:** Socio-demographic and HIV-related characteristics of MAMAS study participants (N = 1777).

Variable	Mean (median, range) or N (%)
Mean age (median, range)	23.58 (22, 18–49)
Mean number of pregnancies (median, range)	3.22 (3, 1–16)
Education, n (%)	
Primary school (elementary) or less	1483 (83.5)
Secondary school (high school) or more	294 (16.5)
Religion, n (%)	
Roman Catholic	331 (18.6)
Seventh Day Adventist	582 (32.8)
Other	862 (48.5)
Luo Ethnicity, n (%)	1641 (92.3)
Currently married, n (%)	1554 (87.5)
Currently living with male partner, n (%)	1546 (87.0)
Male partner has other wives, n (%) (n = 1546)	439 (28.4)
Occupation, n (%)	
Housework	402 (22.7)
Selling things/fish monger	355 (20.0)
Farming/agricultural work/manual labor	742 (41.8)
Male partner’s occupation, n (%) (n = 1545)	
Selling things	172 (11.1)
Farming/agricultural work	586 (37.9)
Fishing	188 (12.2)
Manual labor	243 (15.7)
Household goods, n (%)	
Television	187 (10.5)
Mobile phone	839 (47.2)
Radio	1330 (74.0)
HIV status^1^, n (%)	
Positive	259 (14.7)
Negative	1205 (68.2)
Refused testing	99 (5.6)
Service not available	203 (11.5)
Anticipated male partner stigma related to HIV	627 (35.3)
Anticipated family stigma related to HIV	621 (34.9)
Anticipated stigma from others related to HIV	1051 (59.1)

1 obtained from medical chart after the interview, based on offer of HIV testing during the first ANC visit.

### Health Facility Birth Attitudes

Almost all participants agreed that giving birth at the health facility is safer than at home (97.9%), but there was more variability on items having to do with costs, interpersonal relations, and comfort (Table 2). Of note, 20% of women agreed that women who give birth at health facilities can be tested for diseases without their consent.

**Table 2:** Agreement on the 11 Health Facility Birth Attitude (HFBA) items (N = 1777).

Variable	Agree, n (%)
Giving birth at a health facility is expensive.	823 (48.3)
It is safer for a woman to give birth at a facility than at home.	1738 (97.9)
During a birth, a TBA provides more comfort to a woman than a health worker (doctor or nurse) does.	353 (20.3)
It is difficult to travel to a health facility if a woman goes into labor at night.	825 (46.7)
Women who give birth at health facilities are often treated harshly by health workers	287 (16.7)
A doctor or a nurse is better able to deal with problems that arise during childbirth than a TBA	1719 (97.0)
It is acceptable for a woman to be assisted by a male doctor or nurse during childbirth	1566 (88.6)
TBAs have more flexible arrangements for payment than do health facilities.	558 (32.0)
Women who give birth at health facilities undergo a lot of procedures such as injections, stitches and surgery.	1489 (84.6)
Women who give birth at health facilities may be tested for diseases without their consent.	357 (20.2)
Childbirth goes faster with a TBA	149 (8.6)

After conducting unadjusted analyses to identify significant associations (Table 3), we ran a multivariable model, accounting for clustering by site including items that were significantly associated with the HFBA score, as well as other variables that have been shown to be important for childbirth decision-making in the literature. In these adjusted analyses, women who were married were more likely to have a high HFBA score, as were women with a high score on the HIV knowledge index (Table 4). Women who anticipated HIV-related stigma from their male partner had lower adjusted odds of having positive attitudes about giving birth at the health facility than women who did not anticipate such stigma. Repeating these analyses with a continuous (normally transformed) HFBA variable produced similar results, with anticipated male partner stigma, marital status, and the HIV knowledge index having strong significant adjusted relationships with HFBA, but with occupation and television ownership also retaining significance in the adjusted model (data not shown).

**Table 3:** Unadjusted logistic regression of predictors of health facility birth attitude (n = 1759).

Variable	Low HFBA Score (N = 919)	High HFBA Score (N = 840)	OR (95%CI) for a high HFBA score
Mean age (median, range)	23.64 (22, 18–43)	23.58 (22, 18–49)	0.998 (0.981–1.015)
Mean parity (median, range)	3.27 (3, 1–16)	3.21 (3, 1–12)	0.986 (0.942–1.032)
Education : primary or less vs. (more than) secondary	762 (82.9)	709 (84.4)	1.115 (0.865–1.437)
Religion: 7^th^ Day Adventist vs. other	307(33.4)	266 (31.7)	0.925 (0.758–1.130)
Ethnicity: Luo vs other ethnicities	845 (91.9)	778 (92.6)	1.099(0.773–1.561)
Marital status : currently married vs. notcurrently married	790 (86.1)	753 (89.6)	1.402 (1.049–1.874)*
Currently living with male partner	795 (86.6)	741 (88.2)	0.864 (0.651–1.146)
In a polygamous relation	237 (25.8)	199 (23.7)	0.892 (0.718–1.108)
Occupation: farming vs other occupation	439 (47.9)	376 (44.8)	0.882 (0.731–1.065)
Male occupation (n = 1535): farming vs other occupation	294 (37.0)	288 (38.9)	1.081 (0.880–1.329)
Household utilities:			
Radio	678 (73.8)	640 (76.2)	1.446 (0.868–2.411)
Television	113 (12.3)	73 (8.7)	1.473 (1.080–1.009)*
Cell phone	447 (48.6)	385 (45.8)	1.119 (0.928–1.350)
Mean HIV knowledge index score (n = 1715)(median, range)	0.87 (0.91, 0.27–1)	0.90 (0.91, 0.45–1)	7.917 (3.285–19.080)**
Mean anticipated stigma score (n = 1697)(median, range)	0.32(0.22, 0–1)	0.22 (0.11, 0–1)	0.309 (0.221–0.432)**
Anticipated stigma from male partner^1^	375 (40.8)	245 (29.2)	0.591 (0.483–0.724)**
Anticipated stigma from family^2^	390 (42.4)	228 (27.1)	0.491 (0.400–0.601) **
Anticipated stigma from others^3^	598 (65.1)	444 (52.9)	0.598 (0.490–0.729)**
Mean perceived community stigma score (n = 1690)(median, range)	0.88 (0.89, 0–2.35)	0.79 (0.83, 0–2.39)	0.540 (0.415–0.703) **

1 120 participants refused to answer.

2 87 participants refused to answer.

3 78 participants refused to answer.

* p<.05.

** p<.01.

**Table 4:** Predictors of a Positive Attitude towards Delivery in a Health Facility (high HFBA score) (n = 1636) – multivariate model accounting for clustering by sitea,b.

Variable	Number of women in the category (n)	Adjusted Odds Ratio (OR) for morepositive attitude towards the HF (95% CI)^a^	Adjusted Risk Ratio (RR) for more positive attitude towards the HF (95% CI)^b^
Age (years)	1636	0.988 (0.969–1.007)	0.997 (0.989–1.006)
Primary or less education	1365	0.889 (0.660–1.198)	1.024 (0.873–1.201)
Farmer vs. other occupation	787	0.806 (0.642–1.011)	0.930 (0.783–1.106)
Currently married	1434	1.658 (1.20–2.289)**	1.17 (0.985–1.401)
Having a television	175	0.757 (0.533–1.076)	0.806 (0.756–0.860)**
HIV knowledge index score	1636	10.775 (4.098–28.331)**	3.51 (1.708–7.232)**
Anticipated male partner stigma	618	0.627 (0.505–0.779)**	0.788 (0.704–0.882)**

* p<.05.

** p<.01.

a Odds ratios (ORs) were calculated using mixed effects logistic regression adjusted for clustering by site.

b Risk ratios (RRs) were calculated using binomial regression adjusted for clustering by site.

### Intended Type of Assistance during Delivery

Women with a low HFBA score had five times the odds of saying that they intended to deliver at home than women with a high HFBA score, as compared to women with a high HFBA score (OR 5.15, 95% CI 2.70–9.84). Examination of the individual anticipated stigma items revealed that women who feared bad treatment by health workers if they tested HIV-positive had two times higher odds of intending to deliver at home (OR 2.02, 95%CI 1.09–3.76) and those who anticipated male partner stigma had 1.6 times higher odds (OR 1.69, 95%CI 1.01–2.59). In a multivariable model for intended type of assistance during delivery (Table 5), women who expected to pay for transport or supplies had lower adjusted odds of intending to deliver at home. In contrast, women who had a low HFBA score or a low level of education had significantly higher adjusted odds of intending to have a home delivery.

**Table 5:** Predictors of the Intention to Deliver with a Traditional Birth Attendant (TBA) vs. a Skilled healthcare provider (n = 1562) - multivariate model accounting for clustering by sitea,b,c.

Variable	Number of women in the category (n)	Adjusted Odds Ratio (OR) for theintention to deliver with a TBA (95% CI)^a^	Adjusted Risk Ratio (RR) for the intention to deliver with a TBA (95% CI)^b^
Primary or less education	1301	4.944 (1.116–21.903)*	4.104 (1.503–11.201)**
Farmer vs. other occupation	742	1.359 (0.726–2.543)	1.290 (0.594–2.800)
Polygamous relationship	390	1.073 (0.593–1.943)	1.001 (0.788–1.274)
Having a television	169	0.675 (0.182–2.51)	0.733 (0.283–1.902)
Expects to pay for transport	672	0.203 (0.080–0.512)**	0.247 (0.090–0.680)**
Expects to pay for supplies	1091	0.087 (0.043–0.174)**	0.140 (0.056–0.350)**
Low HFBA score	820	5.559 (2.686–11.506)**	6.001 (3.847–9.361)**
Anticipated male partner stigma	591	1.205 (0.683–2.125)	1.330 (1.157–1.529)**

* p<.05.

** p<.01.

a Odds ratios (ORs) were calculated using mixed effects logistic regression adjusted for clustering by site.

b Risk ratios (RRs) were calculated using binomial regression adjusted for clustering by site.

c Analysis includes women who expressed an intention to deliver with a TBA or a skilled healthcare provider.

We also examined responses to another variable in our dataset, “HIV-positive women who give birth at health facilities are likely to have their HIV status revealed to their husband/partner or others in the community” (agree or disagree). Overall, 57% of women agreed with this item. This variable was significantly associated with intention for home birth (p = .001), with those women agreeing with this statement being more likely to intend a home birth. In stratified analyses we found that for those women who anticipated male partner stigma, this variable was significantly associated with intention for home birth (p = .001), whereas for those who did not anticipate male partner stigma, there was not a significant association (p = .448).

### Mediation Analyses

**Figure 1:**
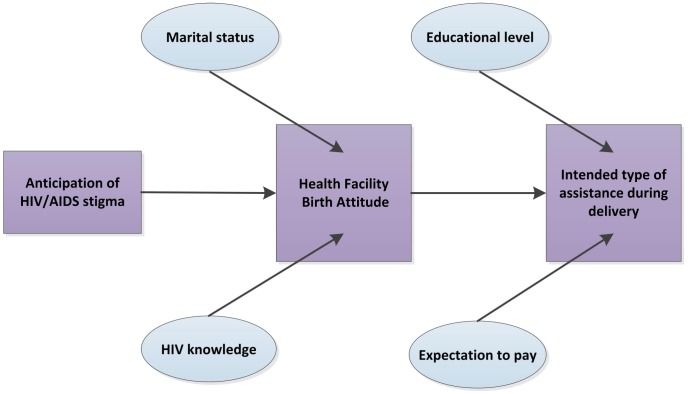
Relationships among HIV-related stigma, HFBA and intended type of assistance during delivery.

We conducted exploratory mediation analyses to examine if the effects of anticipated male partner HIV-related stigma on intended type of delivery might potentially operate through effects on health facility birth attitudes. In unadjusted analysis, anticipating stigma from a partner had an odds ratio of 1.62 (p = .045) for intending to deliver with a TBA. After adjusting for HFBA, the odds ratio decreased to 1.35 and became non-significant (p = .22). This suggests that HIV-related stigma may not directly influence the intended type of assistance during delivery, but operates indirectly as an important factor shaping attitudes toward giving birth at the health facility (Figure 1).

## Discussion

Both women’s attitudes about giving birth in a health facility and their anticipations of HIV-related stigma may be key factors in decision-making for the type of assistance for delivery in high HIV prevalence settings. We found that among pregnant women in rural Kenya, anticipations of HIV-related stigma were associated with a less positive attitude towards giving birth at the health facility. Data from this study, as well as from other studies in East Africa, support the idea that this is linked to women’s perception of the lack of confidentiality at the health facility, resulting in fears that their male partner, family, and community members will learn about their HIV testing and serostatus; potentially resulting in stigma, discrimination, and even violence[40]–[42].

Furthermore, our analyses showed that women’s attitude towards giving birth at a health facility (as measured by the HFBA) was strongly associated with the woman’s intended place of childbirth. Although we cannot make causal inferences based on these cross-sectional data, our analyses suggest that that HIV-related stigma may be an important factor influencing women’s feelings about giving birth at the health facility. As we show that women’s attitudes towards the health facility strongly influence their intentions regarding where they will give birth, reducing HIV-related stigma might tilt the balance in favor of more positive attitudes towards facility-based childbirth.

In addition to the role of HIV-related stigma, we found that women who were currently married had a more positive attitude towards facility-based childbirth than women who were not. Unmarried pregnant women may have a negative attitude towards facility-based childbirth if they perceive that healthcare providers have negative attitudes toward sexual activity and childbearing among single women [43]. It is notable that women who expected to pay for transport and/or supplies related to childbirth had lower adjusted odds of intending to deliver at home, in a setting where lack of money is often mentioned as a barrier for delivering at the health facility. This expectation is therefore most likely not a measure of the ability to pay these costs, but of how well-informed women are about the expenses related to delivering at a health facility.

While at least one ANC visit is the norm for most women in sub-Saharan Africa and acceptance of HIV testing in ANC has increased greatly in many settings due to health worker training and opt-out approaches to HIV testing [23], [44], childbirth in a health facility is still relatively uncommon in rural Kenya. In a similar setting in Tanzania, researchers found that health workers’ emphasis on PMTCT during ANC visits resulted in neglecting to communicate the importance of delivering at the health facility for both HIV negative and positive women [45]. In our qualitative data from this rural Kenyan setting, we learned that many people view giving birth in a health facility as only necessary for women who have serious complications (like HIV) and thus those who give birth in health facilities may run the risk of being assumed to be HIV-positive and suffering from HIV-related stigma [46]. Another effect of emphasizing HIV during ANC visits may be that it increases awareness among women and thereby increases fears of disclosure and stigma.

The current study has several limitations. We only interviewed women who visited the ANC by their seventh month of pregnancy. It is possible that women who do not visit the health facility at all or visit only very late in pregnancy have more negative attitudes towards giving birth at the health facility. However, it would not have been feasible to locate and conduct this research with a community-based sample of pregnant women. Given that 92% of all pregnant women have at least one ANC visit during pregnancy in rural Nyanza [3], our sample is relatively representative. Additionally, we were only able to show a relationship between HIV-related stigma, health facility birth attitudes and the *intended* type of assistance during delivery with these data, but not with the actual type of assistance. It might well be that factors such as lack of decision-making autonomy, lack of money, or the difficulty of transport are more important barriers, and that intentions are not related to actual behavior. However, in longitudinal follow-up of a sub-sample of these women, of those who intended to deliver at home 83% actually did deliver at home, compared to 64% of those who did not intend to deliver at home (p = .055) [47]. Thus women who specifically state that they intend to give birth at home are in fact highly likely to do so. Another limitation is the moderate level of the internal-consistency reliability of the new 11-item HFBA score in this sample. However, this disadvantage is balanced by the strong grounding of the scale items in the cultural context based on qualitative data, as well as the predictive power of this scale vis-a-vis the outcome variables we examined. Although we chose to dichotomize this variable at the median for our primary analyses for ease of interpretation, we recognize that this resulted in loss of information and reduced statistical power. Futhermore, although we adjusted for many important factors in our analyses, there is still the possibility that some other unmeasured confounding variable is responsible for the significant associations we found. Finally our mediation analyses were only exploratory, as they were based on cross-sectional data and we did not adjust for potential confounders between the mediator and the outcome.

### Conclusions

Our study supports the combination of strategies to reduce HIV transmission and prevent maternal and infant mortality [1]. In particular, our findings suggest that working with maternity care providers to increase confidentiality and reduce HIV-related stigma and discrimination, as well as involvement of male partners, could have beneficial effects on women’s utilization of facility-based childbirth services. Interventions for HIV-related stigma reduction might not only reduce stigma but may also result in more women choosing to deliver with a skilled healthcare provider in a health facility. This in turn could contribute to reducing the level of unsafe births and maternal/infant mortality, as well as providing more opportunities for PMTCT. We suggest that future research should further investigate the important role of HIV-related stigma in decision-making around place of delivery in rural settings in Sub-Saharan Africa.
